# Cognitive performance in pain is predicted by effort, not goal desire

**DOI:** 10.1371/journal.pone.0258874

**Published:** 2021-11-04

**Authors:** Jayne Pickering, Nina Attridge, Matthew Inglis

**Affiliations:** 1 Centre for Mathematical Cognition, Loughborough University, Leicestershire, United Kingdom; 2 Department of Psychology, University of Portsmouth, Portsmouth, United Kingdom; Julius-Maximilians-Universität Würzburg, GERMANY

## Abstract

**Background:**

Pain’s disruptive effects on cognition are well documented. The seminal goal-pursuit account of pain suggests that cognitive disruption is less likely if participants are motivated to attended to a focal goal and not a pain goal.

**Objectives:**

Existing theory is unclear about the conceptualisation and operationalisation of ‘focal goal’. This study aims to clarify how goals should be conceptualised and further seeks to test the theory of the goal-pursuit account.

**Methods:**

In a pre-registered laboratory experiment, 56 participants completed an arithmetic task in high-reward/low-reward and pain/control conditions. Pain was induced via cold-water immersion.

**Results:**

High levels of reported effort exertion predicted cognitive-task performance, whereas desire for rewards did not. Post-hoc analyses further suggest that additional effort in the pain condition compensated for pain’s disruptive effects, but when this extra effort was not exerted, performance deficits were observed in pain, compared to control, conditions.

**Conclusion:**

Results suggest that ‘motivation’, or commitment to a focal goal, is best understood as effort exertion and not as a positive desire to achieve a goal. These results solidify existing theory and aid researchers in operationalising these constructs.

## Introduction

Pain may detriment attention and working memory [1–3], which underlie many important daily activities. However, cognitive interruption appears to be unreliable and potentially varies across individuals and/or tasks [4,5]. It is unclear to what extent this disruptive effect is automatic and inevitable and to what extent it can be overcome with conscious will and effort.

The goal-pursuit account argues that commitment to a non-pain goal (e.g. performing well on an arithmetic task) might result in prioritising the non-pain-goal information and reducing accessibility to pain-processing information [6,7]. Alternatively, the motivation-decision model of pain [8] suggests that pain should be conceptualised as a homeostatic state. As such, receptors detect an imbalance (i.e. noxious sensation) then promote an aversive emotional response (i.e. motivation) to restore homeostasis. Since individuals have a limited range of behaviours which can be simultaneously engaged, a homeostatic conflict must be resolved by facilitating/inhibiting one of the conflicting drives. These two accounts are compatible and both explain cognitive disruption through motivation-based mechanisms.

Clearly, there are good theoretical reasons for investigating the interaction of motivation (here defined as ‘the desire to achieve a non-pain goal’) and pain on cognitive-task performance. A straightforward way of manipulating motivation is through financial incentives. In previous research, financial rewards have increased pain threshold and tolerance times for the cold-pressor task [9] and cognitive-task performance benefits when those naturally in pain are offered performance-based rewards [10]. However, few studies have investigated the interplay between rewards, motivation and pain on cognitive-task performance. We designed this study to investigate whether ‘desire to achieve a non-pain goal’ (motivation) predicted cognitive-task performance when in pain. We also investigated the alternative (post-hoc) hypothesis that ‘the amount of resources mobilized to achieve a goal’ (effort) better predicted cognitive-task performance.

The post-hoc hypothesis that pain increases the effort needed to complete a task, rather than lowering motivation to try is a slight reconceptualization of the goal-pursuit account [7], which discusses attentional allocation in terms of prioritising resources and not the level of resources required. The notion of effort exertion also slightly reconfigures/recontextualises Fields’ account [8], which describes a decision to attend to one of multiple conflicting drives, therefore framing motivation as a binary decision rather than a continuous measure. However, it is intuitive that effort affects performance. Furthermore, effort appears to be uni-dimensional, whereas, ‘desire to achieve a goal’ is multi-dimensional. As Van Damme et al. indicated in their goal-pursuit account, the interaction of multiple competing goals may influence behaviour. Somebody who wants to be pain-free/achieve a non-pain reward may not translate that desire into goal-directed action if they feel that goal is impossible or the effort is not justified.

In fact, Motivational Intensity Theory [11] explicitly states that the effort to achieve a goal will increase as goal desirability increases and as task difficulty increases–but only so far as a goal is desirable and deemed achievable. For example, a person will only try hard to get arithmetic questions correct if they both feel that the reward is worth their effort and if they also believe they have the capacity to get the correct answers. If the task is too difficult, participants will exert less effort and ‘give up’. Therefore, the relationship between ‘motivation to achieve a goal’ (desire for financial rewards) and a goal’s outcome (number of correct arithmetic questions) is mediated by increased effort, but this relationship is also underscored by the difficulty of the task and an overall assessment of how achievable the goal is. Although effort ratings were originally included in this study as a way of validating the motivation-goal relationship, Motivational Intensity Theory would predict that effort exertion directly predicts task performance.

Overall, motivation to attend to/distract oneself from pain may have important clinical consequences for adherence to pain-treatment programmes, as well as underlying pain’s cognitive-interruption mechanism (i.e., the extent to which pain’s effects are mediated through environmental features) [12]. Therefore, we explored whether financial rewards can boost motivation, and in turn, whether this boost can overcome pain’s disruptive effects on arithmetic. We predicted similar performance across pain and control conditions with high-rewards, but performance differences between pain and control conditions with low-rewards. We also measured subjective ratings of effort exertion. These were used to assess the alternative, post-hoc, hypothesis that it is effort which predicts performance.

## Method

### Participants

A power calculation for the interaction term of an ANOVA determined the sample size (Cohen’s *f* of .2 (η^2^ of .040), alpha level of .05, and 90% power) of 56 participants. Participants were undergraduate or self-funded postgraduate students from Loughborough University; we specifically recruited from this student population in an attempt to test participants with a strong desire for financial rewards. The participants were aged 18–27, with an average age of 20.5 years (± 2.0), 50% of the sample were female and 82% were right-handed. All participants verified that they were pain free, had not taken any pain killers for at least four hours and did not suffer with chronic pain. Participation was voluntary, all participants signed an informed consent form prior to testing, and study procedures were approved by Loughborough University’s Ethical Approvals (Human Participants) sub-committee (generic proposal G17-P7, sub-proposal C19-40).

### Design

In a repeated-measures design, participants completed an arithmetic task under high- and low-reward and pain- and no-pain conditions. Control blocks alternated with pain blocks. Control blocks did not begin until the participants described themselves as “pain-free” (to prevent pain carryover effects). Participants were counterbalanced so a quarter began with each of the four unique conditions (13 participants per counterbalanced order). Participants completed two consecutive blocks with the same reward type, before alternating to the next reward type. For example, high-rewards pain, high- rewards control, low- rewards pain, then low-rewards control.

### Measures

#### Arithmetic task

In the main task, 180 questions were split across 12 blocks (15 questions per block). Each question asked participants to multiply a single digit by a double-digit. The single digits spanned the numbers 2–6 and the double digits spanned 13–32 (omitting the numbers 20 and 30). This produced 90 unique questions (each presented twice, see below). These unique questions were sorted into six blocks where question difficulty was evenly distributed (according to the size of the numbers, and the presence of carry operations and allowing for special cases) [13]. Using double-digit numbers beyond 12 reduced the likelihood that participants retrieved rote-learned answers–as, within the UK, it is customary to teach times-tables up to 12 × 12 [14].

These six-question lists were then duplicated so two ‘opposite’ blocks would have the same randomized list of questions, e.g., ‘pain with low-rewards’ versus ‘control with high-rewards’. Repeated question lists were always at least three blocks after the original and the order of the questions was randomized within each block.

Each question was presented for 4.5 seconds. Participants typed their answer in a free-text box. A black screen flashed for 200ms after each trial. This was to alert participants that the next trial was beginning (should they have their head bowed towards the keyboard, rather than looking at the screen). Each block was therefore 70.3 seconds long.

#### Financial incentives

Motivation was manipulated through financial incentives: with large-reward blocks (‘pound blocks’) and low-reward blocks (‘penny blocks’). Penny blocks were chosen as the low-motivation option (as opposed to no-reward blocks) because low rewards worsen performance in comparison to no reward at all [15].

Participants were told that they could win a maximum of £15 across the study’s pounds blocks and a maximum of 90p across the penny blocks. They saw a list of nine things/experiences that £15 could buy (but not for 90p) and were asked to reflect on what they would purchase if they won £15 and 90p. (This was an attempt to make the money more concrete and more desirable.)

In penny blocks, participants earned a penny for every correct answer. In pound blocks, the rewards increased in 50p increments for every three questions correct, with a maximum of £2.50 per block.

#### Visual analogue scales

After each block of questions, participants answered “How much pain were you in during the block?” by clicking on a 100-point visual analogue scale (VAS) anchored with “No pain at all” on the left and “Worst pain imaginable” on the right.

Four effort scales were presented on 100-point VAS at the end of the study asking participants to retrospectively evaluate how much effort they exerted in each condition. Each scale said, “I tried my best to answer the questions in the cold-penny/ cold-pound/ warm-penny/ warm-pound blocks” and were anchored with “Not at all” to “Very much so.”

At the start of the study, before the arithmetic task, participants rated their desire to earn the maximum pound and penny amounts, on two 100-point VAS, which asked, “How much would you like to earn [£15/90p]?” and were anchored with “Not at all” and “Very much so.”

#### Water baths and pain induction

Pain was induced through cold-water immersion. Participants submerged their hand in 8-litre water baths (up to their upper palm/lower wrist). For the painful-water bath, the temperature was cooled by a Grant™ immersion cooler and was maintained at 8°C (+/- 1°C), whilst the control-water bath was maintained at 30°C (+/- 1°C). For both water baths, water was circulated by Techne™ thermoregulators. We chose 8°C for the cold temperature as previous research suggests this temperature is far below the 15°C needed to induce pain [16,17], while having the twin advantages of lowering dropout rate and allowing for the hand to be more easily re-warmed in-between blocks (compared to colder temperatures). Rewarming the hand is important as a rapid drop in temperature is perceived as more painful than a constant coldness, because nerves are sensitive to the change-rate in temperature [18,19].

### Procedure

Study information and a pre-screening medical questionnaire were shown to participants in advance of the session. At the beginning of the session, the experimenter checked participants’ eligibility, took written consent, and collected demographic information (age, sex, handedness).

Participants completed five practice trials of the arithmetic task (without their hand submerged) and were given instructions for answering the VAS. Participants completed the desire-for-rewards VAS before beginning the main task. Participants completed twelve blocks of the arithmetic task with a one-minute break in between blocks.

100-point VAS pain ratings were completed at the end of every block, then their performance was displayed on the screen. Participants were given their performance-based allowance at the end of every block to convince any sceptical participants that they would earn money based on their performance. The money was laid out on two separate sheets of paper labelled ‘pound blocks’ and ‘penny blocks’ so they could see how their rewards were accruing. After the task, participants rated their effort across the four conditions on four VAS scales.

### Pre-registration, hypotheses and data access

In support of open science practices, we pre-registered our hypotheses, sample size and analysis plans prior to data collection. Pre-registered materials can be found at www.aspredicted.org (#24223).; an anonymised PDF for peer review is available at https://aspredicted.org/blind.php?x=67bt96. Any a posteriori analyses, which were not pre-registered, are labelled as exploratory below. We hypothesized that pain would interact with financial-incentive condition (i.e. motivation) to affect arithmetic performance. All analyses were two-tailed. Data files and analysis scripts are openly available at Figshare: https://doi.org/10.6084/m9.figshare.13703407 and https://doi.org/10.6084/m9.figshare.13703860.

## Results

### Data cleaning

We pre-registered six exclusion (and replacement) criteria. 1) Participants who did not complete the experiment (four participants removed, two quit after the practice questions and two could not endure the pain). 2) Participants whose average pain-block pain ratings did not significantly differ from their average control-block pain ratings (checked via paired-samples *t*-tests on each participants’ data; no participants removed). 3) Participants who were more than 2.5 standard deviations from the group mean, across all blocks–to reduce skew (no participants removed). 4) Participants whose average was below 27% (4/15 questions per block) in the control blocks only–to control for floor effects (two participants removed). 5) Participants whose average was over 95% (averaging across all blocks in all conditions) and also 90% or over correct in every condition type–to control for ceiling effects (three participants removed). 6) Participants who rated their desire for £15 to be less than 25 points higher than their desire for 90p –used as a manipulation check, but only applied if six or fewer participants met it (met by 26 participants; no participants removed). After these checks, five complete data sets were removed, and participants were replaced before the analysis began.

### Pre-registered analyses

**Fig 1:**
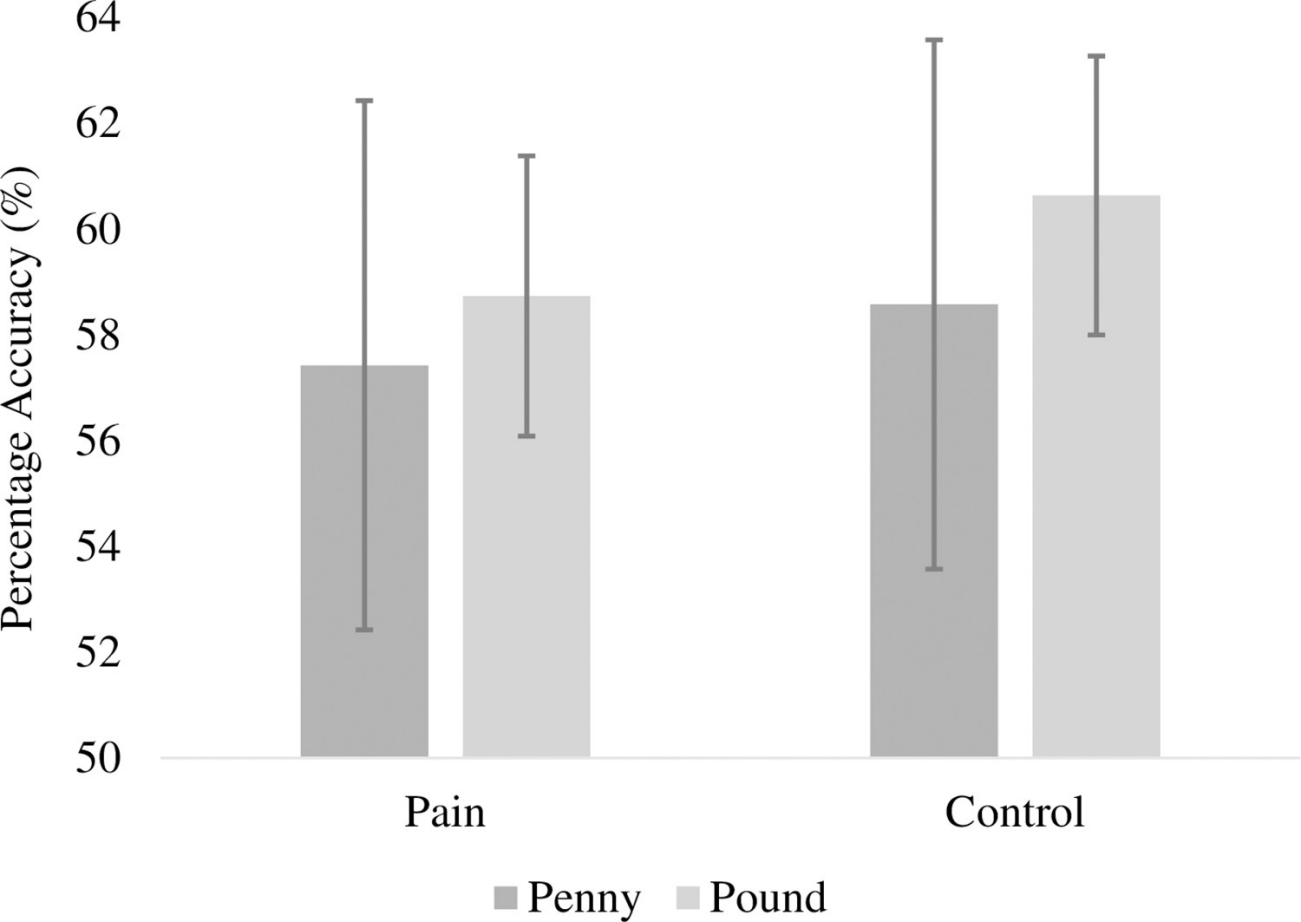
Percentage accuracy by pain and incentive conditions. Error bars show ± 1 standard error of the mean.

A 2 (pain: pain, no pain) ×2 (motivation condition: low incentives, high incentives) repeated-measures ANOVA was conducted on percentage-accuracy arithmetic scores. As predicted, there was a significant main effect of motivation condition, with better performance in the high-motivation condition (59.7% ± 19.1%; M±SD) than the low-motivation condition (58.0% ± 19.3%; M±SD), *F*(1,55) = 5.12, *p* = .028, η_p_^2^ = .085. There was a non-significant main effect of pain, with similar performance in the pain (58.1% ± 19.5%; M±SD) and no-pain conditions (59.6% ± 19.0%; M±SD), *F*(1,55) = 3.44, *p* = .069, η_p_^2^ = .059. Against predictions, there was not a significant interaction, *F*(1,55) = 0.12, *p* = .731, η_p_^2^ = .002. These data are shown in Fig 1. Post-hoc tests indicated that accuracy in the high-incentive conditions was similar across pain and control conditions, *t*(55) = -1.40, *p* = .168, *d* = 0.10. However, the accuracy in the low-incentive conditions was also similar across pain and control conditions, *t*(55) = -0.84, *p* = .404, *d* = 0.06.

### Exploratory post-hoc analyses

To assess whether participants wanted the pound-block rewards significantly more than the penny-block rewards, a paired-sample *t*-test was conducted, which showed a significant difference between the desire-for-£15 VAS ratings (90 ± 15; M±SD) compared to the desire-for-90p VAS ratings (58 ± 29; M±SD), *t*(55) = 8.36, *p* < .001, *d* = 1.42.

**Fig 2:**
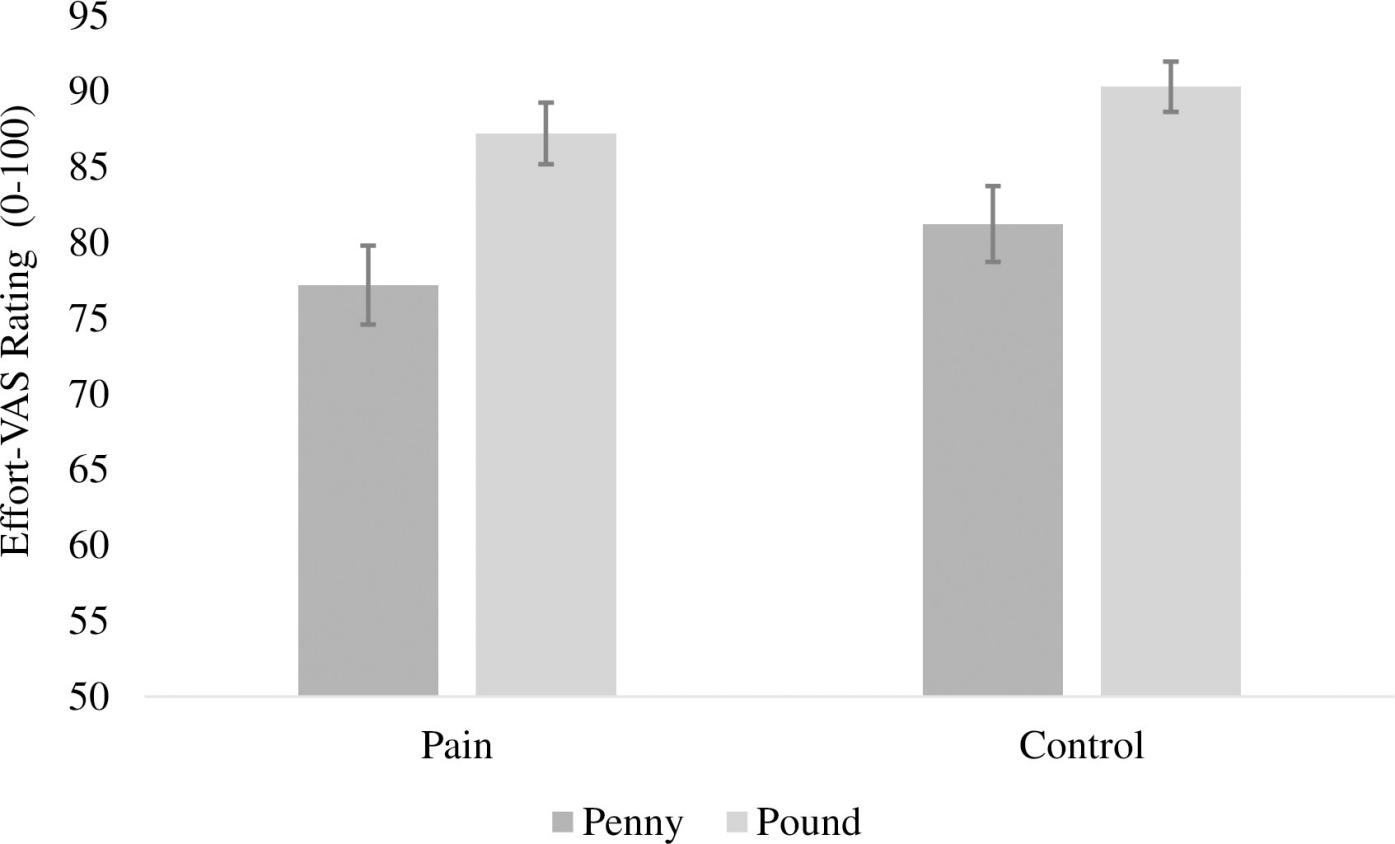
VAS-rated effort in pain and incentive conditions. Error bars show ± 1 standard error of the mean.

To see if different amounts of effort were exerted across the different conditions, we conducted a 2 (pain: pain, no pain) ×2 (motivation condition: low, high) repeated-measures ANOVA on participants’ effort-VAS ratings. Interestingly, the results, and effect sizes, follow the same pattern as the percentage-accuracy analysis. There was a significant main effect of motivation condition, with more effort exerted in the pound blocks (88.8% ± 12.0%; M±SD) than the penny blocks (79.2 ± 16.7%; M±SD), *F*(1,55) = 25.08, *p* < .001, η_p_^2^ = .313. There was a non-significant main effect of pain, with similar effort in the pain (82.2% ± 15.6%; M±SD) and no-pain conditions (85.8% ± 13.7%; M±SD), *F*(1,55) = 3.33, *p* = .074, η_p_^2^ = .057. The interaction, shown in Fig 2, was non-significant, *F*(1,55) = 0.22, *p* = .645, η_p_^2^ = .004. This suggests that offered incentives, and not the pain condition, may have driven effort exertion.

Further analyses were conducted to see how motivation and effort affected performance. Firstly, each condition’s effort rating was correlated with its accuracy. The correlation was significant for pain-pound blocks, *r*(55) = .53, *p* < .001; pain-penny blocks, *r*(55) = .43, *p* = .001; and control-pound blocks, *r*(55) = .41, *p* = .002; but non-significant for control-penny blocks, *r*(55) = .26, *p* = .0505. Effort was near ceiling for the pound blocks (especially control-pound) but was more varied for penny blocks. However, desire-for-90p VAS did not correlate with average penny-condition accuracy, *r*(55) = .08, *p* = .572, and desire-for-£15 VAS did not correlate with average pound-condition accuracy, *r*(55) = −.08, *p* = .576. This suggests that self-reported effort predicts performance better than motivation (the desire to achieve a non-pain goal).

Finally, to test the hypothesis that effort and motivation are separate constructs, we calculated two correlation coefficients between desire-for-90p VAS with an averaged VAS-effort rating for penny conditions, *r*(55) = .17, *p* = .206, and desire-for-£15 VAS with an averaged VAS-effort rating for pound conditions, *r*(55) = .06, *p* = .660.

In sum, these data suggest that effort and motivation are separate constructs; only self-reported effort predicts performance in and out of pain, whereas self-reported motivation does not.

### Post-hoc sub-group analyses

We wanted to know whether a disruptive effect of pain on cognition can be overruled by incentives. Since there was no disruptive effect of pain in these data, we created a sub-group of participants who were adversely affected by pain (via a median split on control arithmetic minus pain-arithmetic difference scores) and re-ran the main analysis. A 2 (pain: pain, no pain) ×2 (motivation condition: low incentives, high incentives) repeated-measures ANOVA was conducted on percentage-accuracy arithmetic scores. This forced a main effect of pain, with better performance in the control condition (60.9 ± 17.8%; M±SD) than the pain condition (55.0% ± 17.6%; M±SD), *F*(1,28) = 42.70, *p* < .001, η_p_^2^ = .604. However, there was not an effect of motivation, with similar performance in the penny (57.0% ± 17.7%; M±SD) and pound conditions (58.9% ± 17.9%; M±SD), *F*(1,28) = 2.50, *p* = .125, η_p_^2^ = .082. The predicted interaction was non-significant, *F*(1,28) = 0.09, *p* = .772, η_p_^2^ = .003.

It is probable that where exerted effort differs across pain conditions, performance will also differ across pain conditions. Therefore, a median split on effort-difference scores was used to create a sub-group of participants who tried harder in the pain conditions compared to the control conditions, and a paired-sample *t*-test compared performance across conditions. There was no effect of pain condition on performance, *t*(26) = .18, *p* = .862. However, for the sub-group of participants who tried harder in the control conditions compared to the pain conditions, a paired-sample *t*-test indicated better performance in the control condition (55.4% ± 18.7%; M±SD) compared to the pain condition (52.6% ± 19.1%; M±SD), *t*(28) = 2.45, *p* = .021. This suggests that compensatory effort in the pain condition may nullify the disruptive effect of pain on performance but a lack of compensatory effort may result in a performance difference between conditions.

## Discussion

Participants completed arithmetic questions in pain and control conditions, receiving either low incentives or high incentives for accuracy. Participants rated their desire for £15 as being greater than their desire for 90p. Participants also rated their exerted effort levels to be higher for pound blocks than penny blocks, and performance was better on pounds blocks compared to penny blocks. Collectively, this suggests that motivation was successfully manipulated.

However, the desire for incentives did not correlate with performance. This is, perhaps, unsurprising. People have cognitive limitations and willing themselves to perform well will only improve their performance by so much. A more nuanced metric may be useful for future studies, which balances the desire for money with a realistic appraisal of success and accounting for competing goals.

With motivation successfully manipulated, we predicted similar performance between pain-pound and control-pound conditions, believing that participants would exert maximal effort in the high-reward conditions and overcome pain’s disruptive effects. Although there was similar performance across these conditions, there was also similar performance between the pain-penny and control-penny conditions, where motivation to try was lower. Therefore, the main theoretical prediction that extra motivation can overcome pain’s disruptive effects was not supported. Instead, the results only showed a trend that accuracy may be lower in the painful conditions compared to control conditions, but this was not statistically significant.

However, financial incentives affected exerted-effort levels and task performance in similar ways. This suggests that incentives’ effects on task performance may be mediated through increased effort exertion. Effort exertion has the more direct impact on performance and significantly correlated with task performance across three conditions (with a lower, but still positive, correlation in the fourth), whereas the desire for incentives did not predict performance in either the high- or low-incentive conditions. Furthermore, post-hoc sub-group analyses suggested that compensatory effort in the pain condition may nullify the disruptive effect of pain on performance but a lack of compensatory effort may result in a performance difference across conditions. Measures of effort continuously predicted arithmetic-task performance, whereas measures of motivation did not. Even in post-hoc sub-group analyses, where only those participants who were adversely affected by pain were considered, we did not find a compensatory effect for incentives nullifying the disruptive effect of pain on performance. Additionally, desire for incentives and effort exertion did not correlate, further suggesting that these are distinct constructs.

Although these results suggest an important role for effort when overcoming pain’s cognitive disruption, there are several reasons to interpret these data cautiously. Firstly, participants’ performance was transparent to them and so their effort ratings may reflect demand characteristics. In other words, it may be that participants simply remembered how well they performed and reported their effort accordingly. Future studies would benefit from making participants’ scores opaque to them (e.g., by using reaction-time tasks or more complicated questions). As there are good theoretical reasons to believe that effort overcomes pain’s disruptive effects [11], there is no reason to assume that demand characteristics explain these data; however, this is an important issue which future research needs to investigate.

A second limitation of this study is that effort ratings were only taken at the end of the experiment, and not after every block. Taking effort ratings at the end of the study may limit the demand characteristics detailed above. However, they may also make the effort ratings more vulnerable to memory error. Since the effort-VAS measures correlated so strongly with performance, we think it unlikely that a memory bias has diluted these measurements. However, more immediate effort ratings may improve the internal validity of this measures and should be considered in future research.

A third limitation is that effort was not directly manipulated and is a self-report measure. Multiple metrics of effort, or objective physiological measures of effort, would be beneficial and improve the internal validity of this measurement. However, it is not straightforward to measure effort in a physiological sense. For example, Cancela and Silvestrini [20] used multiple metrics to measure effort: cardiac pre-ejection period (PEP), PEP-Lozano (a corrected metric for PEP), diastolic blood pressure, systolic blood pressure, and heart rate. They found differences between these measures, prompting questions about which measure is best. The authors found that PEP-Lozano-by-time interactions best predicted their data, but this complicates the use of cold-induced pain, which tends to change throughout a block as the body acclimatises to the temperature [19]. Overall, there is only an issue with internal validity if we think it is likely that subjective self-report measures of effort do not somehow measure ‘the amount of resources mobilized to achieve a goal’. As we are interested in *conscious and deliberate* exertion of effort, we would expect participants to be aware of their effort exertion. Nevertheless, future research would benefit from using subjective effort ratings when task difficulty has also been manipulated, as a way of validating self-report measures of effort.

With these limitations in mind, this study offers preliminary data on a new, slightly reconfigured, hypothesis. As with all theories, multiple studies will be needed to develop a substantive evidence base to support its hypotheses. If the ‘desire to achieve a goal’ and ‘effort expenditure to achieve a goal’ are two separate constructs, then this study suggests that the operationalisation of ‘motivation’ or ‘commitment to a focal goal’ is best achieved through effort ratings and not desire-for-outcome ratings. This is an important theoretical step as both the goal-pursuit account [7] and motivational-decision theory [8] implicitly suggest that motivation may be best understood as the desire to achieve a particular goal.

This reconfiguration of ‘motivation’ towards ‘effort’ may explain why pain has such an unreliable effect on cognition. Exerted effort was high across the study and was not significantly different between pain and control conditions. This may explain why no detrimental effects of pain were found. It is probable that where exerted effort differs across pain conditions, so too will performance across pain conditions. The unreliability of pain’s disruptive effect is well-documented [4,5]. It is therefore essential to look at moderating factors on pain’s disruptive effects. Therefore, measuring exerted effort in future pain-disruption studies may help to explain both null and significant findings, representing both an important theoretical and empirical advancement.

Overall, this study successfully manipulated ‘motivation to try’ through monetary incentives. Although the use of incentives did not interact with the pain condition, it has established that motivational boosts can be achieved quickly and easily. In future research, if intrinsic motivation to succeed can be lowered, the extrinsic motivators may have more impact. This study’s attempt to tease apart ‘motivation to try’ and ‘effort exertion’ cautiously offers a preliminary, but promising, new line of research.

## References

[1] Berryman C, Stanton TR, Bowering JK, Tabor A, McFarlane A, Lorimer Moseley G. Evidence for working memory deficits in chronic pain: a systematic review and meta-analysis. Pain. 2013;154(8):1181–96. doi: 10.1016/j.pain.2013.03.002 23707355

[2] Berryman C, Stanton TR, Bowering KJ, Tabor A, McFarlane A, Moseley GL. Do people with chronic pain have impaired executive function? A meta-analytical review. Clin Psychol Rev. 2014;34(7):563–79. Available from: doi: 10.1016/j.cpr.2014.08.003 25265056

[3] Moriarty O, McGuire BE, Finn DP. The effect of pain on cognitive function: a review of clinical and preclinical research. Prog Neurobiol. 2011;93(3):385–404. Available from: doi: 10.1016/j.pneurobio.2011.01.002 21216272

[4] Attridge N, Eccleston C, Noonan D, Wainwright E, Keogh E. Headache impairs attentional performance: a conceptual replication and extension. J Pain [Internet]. 2017;18(1):29–41. Available from: doi: 10.1016/j.jpain.2016.09.007 27742412

[5] Moore DJ, Keogh E, Eccleston C. The interruptive effect of pain on attention. Q J Exp Psychol. 2012;65(3):565–86. doi: 10.1080/17470218.2011.626865 22136653

[6] Schrooten MG, Van Damme S, Crombez G, Peters ML, Vogt J, Vlaeyen JW. Non-pain goal pursuit inhibits attentional bias to pain. Pain. 2012;153(6):1180–6. doi: 10.1016/j.pain.2012.01.025 22409943

[7] Van Damme S, Legrain V, Vogt J, Crombez G. Keeping pain in mind: a motivational account of attention to pain. Neurosci Biobehav Rev. 2010;34:204–13. doi: 10.1016/j.neubiorev.2009.01.005 19896002

[8] Fields H. A motivation-decision model of pain: the role of opioids. In: Flor H, Kalso E, Dostrovsky JO, editors. Proceedings of the 11th World Congress on Pain. Seattle, USA: IASP Press; 2006. p. 449–59.

[9] Bouaziz N, Moulier V, Lettelier-Galle T, Osmond I, Faivre-Wojakiewicz A, Benadhira R, et al. Impact of reward on pain threshold and tolerance to experimental pain (cold pressor task) in healthy subjects and patients with schizophrenia. Psychiatry Res. 2017;254(4):275–8. Available from: 10.1016/j.psychres.2017.04.045. 28482197

[10] Verhoeven K, Crombez G, Eccleston C, Van Ryckeghem DML, Morley S, Van Damme S. The role of motivation in distracting attention away from pain: an experimental study. PAIN. 2010;149(2):229–34. Available from: doi: 10.1016/j.pain.2010.01.019 20188469

[11] Brehm JW, Self EA. The intensity of motivation. Annual review of psychology. 1989 Feb; 40(1):109–31. doi: 10.1146/annurev.ps.40.020189.000545 2648973

[12] Jensen MP. Enhancing motivation to change in pain treatment. In: Turk DC, Gatchel RJ, editors. Psychological approaches to pain management: a practioner’s handbook. 3rd ed. New York, NY: Guilford Publications; 2018. p. 78–111.

[13] De Brauwer J, Verguts T, Fias W. The representation of multiplication facts: developmental changes in the problem size, five, and tie effects. J Exp Child Psychol. 2006;94(1):43–56. doi: 10.1016/j.jecp.2005.11.004 16376370

[14] Department for Education. Multiplication tables check trials to begin in schools [Internet]. 2018 [cited 2019 Jul 18]. Available from: https://www.gov.uk/government/news/multiplication-tables-check-trials-to-begin-in-schools.

[15] Gneezy U, Rustichini A. Pay enough or don’t pay at all. Q J Econ. 2000;115(3):791–810.

[16] Ziegler D, Mayer PE, Gries FA. Evaluation of thermal, pain, and vibration sensation thresholds in newly diagnosed type 1 diabetic patients. Journal of Neurology, Neurosurgery & Psychiatry. 1988 Nov 1;51(11):1420–4. 10.1136/jnnp.51.11.1420 PMC1032813 3236020

[17] Wasner GL, Brock JA. Determinants of thermal pain thresholds in normal subjects. Clinical Neurophysiology. 2008 Oct 1;119(10):2389–95. doi: 10.1016/j.clinph.2008.07.223 18778969

[18] Prescott SA, Ratté S. Somatosensation and pain. In Conn’s Translational Neuroscience 2017 Jan 1 (pp. 517–539). Academic Press.

[19] Wolf S, Hardy JD. Studies on pain. Observations on pain due to local cooling and on factors involved in the “cold pressor” effect. The Journal of clinical investigation. 1941 Sep 1;20(5):521–33. doi: 10.1172/JCI101245 16694857 PMC435082

[20] Cancela T, Silvestrini N. Impact of pain on mental effort assessed as cardiovascular reactivity. Pain reports. 2021;6(1). doi: 10.1097/PR9.0000000000000917 33977185 PMC8104428

